# Prevalence and Strain Characterization of *Clostridioides (Clostridium) difficile* in Representative Regions of Germany, Ghana, Tanzania and Indonesia – A Comparative Multi-Center Cross-Sectional Study

**DOI:** 10.3389/fmicb.2018.01843

**Published:** 2018-08-07

**Authors:** Mwanaisha Seugendo, Iryna Janssen, Vanessa Lang, Irene Hasibuan, Wolfgang Bohne, Paul Cooper, Rolf Daniel, Katrin Gunka, R. L. Kusumawati, Stephen E. Mshana, Lutz von Müller, Benard Okamo, Jan R. Ortlepp, Jörg Overmann, Thomas Riedel, Maja Rupnik, Ortrud Zimmermann, Uwe Groß

**Affiliations:** ^1^Department of Pediatrics and Child Health, Catholic University of Health and Allied Sciences, Mwanza, Tanzania; ^2^Institute of Medical Microbiology, University Medical Center Göttingen Göttingen, Germany; ^3^St. Martin de Porres Hospital, Eikwe, Ghana; ^4^Department of Genomic and Applied Microbiology, University of Göttingen, Göttingen, Germany; ^5^Department of Microbiology, Faculty of Medicine, Universitas Sumatera Utara, Medan, Indonesia; ^6^Department of Medical Microbiology, Catholic University of Health and Allied Sciences, Mwanza, Tanzania; ^7^Institute of Medical Microbiology, Saarland University, Homburg, Germany; ^8^Asklepios Kliniken Schildautal, Seesen, Germany; ^9^Department Microbial Ecology and Diversity Research, Leibniz Institute DSMZ, Braunschweig, Germany; ^10^Institute of Public Health Maribor, Maribor, Slovenia; ^11^Faculty of Medicine, University of Maribor, Maribor, Slovenia

**Keywords:** *Clostridioides difficile*, epidemiology, Germany, Indonesia, Africa, multi-center study, ribotype, toxinotype

## Abstract

*Clostridioides (Clostridium) difficile* infections (CDI) are considered worldwide as emerging health threat. Uptake of *C. difficile* spores may result in asymptomatic carrier status or lead to CDI that could range from mild diarrhea, eventually developing into pseudomembranous colitis up to a toxic megacolon that often results in high mortality. Most epidemiological studies to date have been performed in middle- and high income countries. Beside others, the use of antibiotics and the composition of the microbiome have been identified as major risk factors for the development of CDI. We therefore postulate that prevalence rates of CDI and the distribution of *C. difficile* strains differ between geographical regions depending on the regional use of antibiotics and food habits. A total of 593 healthy control individuals and 608 patients suffering from diarrhea in communities in Germany, Ghana, Tanzania and Indonesia were selected for a comparative multi-center cross-sectional study. The study populations were screened for the presence of *C. difficile* in stool samples. Cultured *C. difficile* strains (*n* = 84) were further subtyped and characterized using PCR-ribotyping, determination of toxin production, and antibiotic susceptibility testing. Prevalence rates of *C. difficile* varied widely between the countries. Whereas high prevalence rates were observed in symptomatic patients living in Germany and Indonesia (24.0 and 14.7%), patients from Ghana and Tanzania showed low detection rates (4.5 and 6.4%). Differences were also obvious for ribotype distribution and toxin repertoires. Toxin A^+^/B^+^ ribotypes 001/072 and 078 predominated in Germany, whereas most strains isolated from Indonesian patients belonged to toxin A^+^/B^+^ ribotype SLO160 and toxin A^-^/B^+^ ribotype 017. With 42.9–73.3%, non-toxigenic strains were most abundant in Africa, but were also found in Indonesia at a rate of 18.2%. All isolates were susceptible to vancomycin and metronidazole. Mirroring the antibiotic use, however, moxifloxacin resistance was absent in African *C. difficile* isolates but present in Indonesian (24.2%) and German ones (65.5%). This study showed that CDI is a global health threat with geographically different prevalence rates which might reflect distinct use of antibiotics. Significant differences for distributions of ribotypes, toxin production, and antibiotic susceptibilities were observed.

## Introduction

*Clostridioides (Clostridium) difficile* infections (CDI) have become an emerging health threat; more than 450,000 cases with approximately 30,000 deaths have been estimated to occur every year in the USA alone (Lessa et al., 2015). Infections with this pathogen may stay asymptomatic or range from mild diarrhea up to pseudomembranous colitis or even develop into life-threatening toxic megacolon (Rupnik et al., 2009). Based on its impact on health systems, *C. difficile* has been prioritized as pathogen of the highest priority group for surveillance and epidemiological research (Balabanova et al., 2011). It is generally believed that only toxigenic strains cause disease with toxin B (TcdB) being most important for virulence. The majority of virulent strains produce toxin A (TcdA) and toxin B (TcdB) simultaneously. Some strains also produce a binary toxin (CDT), either together with TcdA and TcdB or alone (Eckert et al., 2015). Strains with modifications in the toxin A and B coding region, the so-called pathogenicity locus (PaLoc), compared to the well-characterized reference strain VPI 10463, are defined as variant strains. They can be differentiated by toxinotyping, a PCR-restriction fragment length polymorphism (RFLP)-based method (Rupnik and Janezic, 2016). However, for epidemiological purposes, classification of *C. difficile* strains is currently mainly achieved by PCR ribotyping (Wilcox, 2012). Based on phylogenetic analysis eight clades are currently distinguished, and comprise non-toxigenic (PaLoc absent) strains and clades with representative ribotypes for each (Knight et al., 2015). Most epidemiological studies to date have been performed in middle- and high income countries, especially in North-America, Europe, Australia, and to a lesser extend in Asia (Burke and Lamont, 2014). Only very limited data are available from Africa. We therefore previously performed prospective cross-sectional studies in Ghana and Tanzania, which indicated a high prevalence of non-toxigenic strains in sub-Saharan Africa (Seugendo et al., 2015; Janssen et al., 2016). Using the identical study design we also investigated prevalence rates of *C. difficile*-positive individuals in Medan/Indonesia and Seesen/Germany in the similar time period as in Ghana and Tanzania and performed further characterization of the obtained isolates. The aim of this article is to compare the data obtained from Indonesia and Germany with the previously published data from Ghana and Tanzania and to present this multi-center prospective study as a whole in order to compare prevalence rates, strain distribution and characterization of *C. difficile* in these four distinct geographical regions.

## Materials and Methods

### Study Design

The four studies to be compared here were performed during the time period of September 2013 to October 2014 in the St. Martin de Porres Hospital in Eikwe/rural Ghana (Janssen et al., 2016), the Bugando Medical Center and Sekou Toure Regional Hospital in Mwanza/urban Tanzania (Seugendo et al., 2015), the Adam Malik Hospital and Pematang Siantar Hospital in Medan/urban Indonesia, and the Asklepios Hospital Schildautal in Seesen/rural Germany. This study was carried out in accordance with the recommendations of the guidelines set by the University Medical Center Göttingen. The protocol was approved by the Ethical Committees responsible for the participating hospitals and the University Medical Center, Göttingen, Germany (29/3/11). All subjects gave written informed consent in accordance with the Declaration of Helsinki. The participants were classified into two groups; those with diarrhea (symptomatic group) and a non-diarrheic control group, consisting of patients, relatives of the patients without diarrhea or employees of the respective hospitals. Following informed consent, all participants of the study submitted a stool sample that was directly examined for the presence of *C. difficile* at the local laboratory. All examiners were trained beforehand in bacterial identification at the Institute of Medical Microbiology of the University Medical Center Göttingen, Germany.

### Microbiological Analysis

Stool samples were cultured on *C. difficile* selective agar (bioMérieux, Marcy-l’Étoile, France) or chromogenic agar (CHROMagar, Paris, France) and incubated for up to 48 h in an anaerobic jar, using anaerobic packs (bioMérieux). At least five grown *C. difficile* colonies were isolated on COS Columbia blood agar, enriched with 5% sheep blood (bioMérieux) and stored in a microbank system (bestbion dx GmbH, Cologne, Germany) until further analysis (e.g., identification by MALDI-TOF mass spectrometry, toxin and sensitivity test) in Göttingen, Germany.

*Clostridium difficile* identification was verified by MALDI-TOF mass spectrometry (Biotyper, Bruker Daltonics, Bremen, Germany) with score values ≥ 2000. Susceptibility of *C. difficile* isolates to macrolides (erythromycin or clarithromycin), moxifloxacin, metronidazole, and vancomycin was determined with Etest^®^ (bioMérieux) according to standard protocols. Briefly, bacterial isolates were cultured in BHI broth (BD Difco Fraser Broth Supplement, Heidelberg, Germany), adjusted with 0.5% yeast extract and 0.03% L-cysteine and incubated in an anaerobic chamber (Coy Laboratory Products, Michigan, United States) with an atmosphere of 5% CO_2_, 5% H_2_, and 90% N_2_. Bacterial cells were diluted using 0.9% saline to McFarland standard 1 and streaked onto Mueller-Hinton agar supplemented with 5% horse blood + 20 mg/l β-NAD^+^ (bioMérieux). Reproducibility was ensured by inclusion of the control strains *C. difficile* 630 (DSMZ-27543, Leibniz Institute DSMZ, Braunschweig, Germany) and *C. difficile* R 20291 (DSM-27147, Leibniz Institute DSMZ, Braunschweig, Germany). After two days of incubation with Etest^®^ strips (bioMérieux), the minimal inhibitory concentrations (MIC) were determined. Toxin production was investigated using the Serazym^®^ *C. difficile* toxin A+B immunoassay (Seramun Dianostica GmbH, Heidesee, Germany) or Quik Chek Complete (Alere Techlab, Blacksburg, VA, United States). Furthermore, isolates were specified by multiplex PCR to detect toxin gene profiles (Stahlmann et al., 2014) and PCR ribotyped by agarose (isolates from Ghana and Indonesia) or capillary gel electrophoresis (isolates from Germany and Tanzania) according to consensus protocols (CDRN, ECDIS-Net) as previously described (Janezic and Rupnik, 2010; Fawley et al., 2016; van Dorp et al., 2016; Berger et al., 2018).

## Results

### Prevalence Rates of *C. difficile* Carriage

This multi-center, cross-sectional study covers a total of 1,201 participants, 608 inpatients with diarrhea and 593 controls. Prevalence rates of *C. difficile* carriage were highest in the participants from the German and the Indonesian study sites compared with the two African study sites (**Table 1**).

**Table 1:** Prevalence rates of *C. difficile* in the different study sites.

Country	Participants	*C. difficile* positive	Subgroup	*C. difficile* positive	No. of *C. difficile* isolates
Germany	242	29	Symptomatic (*n* = 121)	29 (24.0%)	29
			Control (*n* = 121)	0	0
Indonesia	402	28	Symptomatic (*n* = 170)	25 (14.7%)	30
			Control (*n* = 232)	3 (1.3%)	3
Ghana	307	15	Symptomatic (*n* = 176)	8 (4.5%)	8
			Control (*n* = 131)	7 (5.3%)	7
Tanzania	250	9	Symptomatic (*n* = 141)	9 (6.4%)	7^∗^
			Control (*n* = 109)	0	0
Total	1,201	81	Symptomatic (*n* = 608)	71 (11.7%)	74
			Control (*n* = 593)	10 (1.7%)	10


*^∗^Two isolates were not available for further analysis.*

In fact, *C. difficile* was only found in symptomatic patients but not in asymptomatic controls in the German study site. The mean age of *C. difficile*-positive patients was 73.5 years in comparison to 68.7 years in the *C. difficile*-negative group. Similarly, *C. difficile* was nearly exclusively recovered only from symptomatic patients in Indonesia. In contrast to the situation in Germany, 50% of *C. difficile*-positive and 21.4% of *C. difficile*-negative participants in Indonesia were children younger than 6 years of age.

Like in Seesen/Germany, *C. difficile* was only found in symptomatic patients in the Tanzanian study site but not in the healthy control group. 48.9% of Tanzanian patients with diarrhea were children 12 years of age or younger and five out of seven *C. difficile* isolates were recovered from children (Seugendo et al., 2015). Whereas a clear difference in prevalence rates between symptomatic patients and healthy controls was found in the above-mentioned three study sites, nearly equal colonization rates were found in symptomatic and asymptomatic participants from the Ghanaian study site. Here, the majority of *C. difficile*-positive asymptomatic controls were children aged 0–5 years, whereas the majority of *C. difficile*-positive symptomatic patients aged 16–45 years (Janssen et al., 2016).

### Ribotypes of *C. difficile* Isolates

**FIGURE 1:**
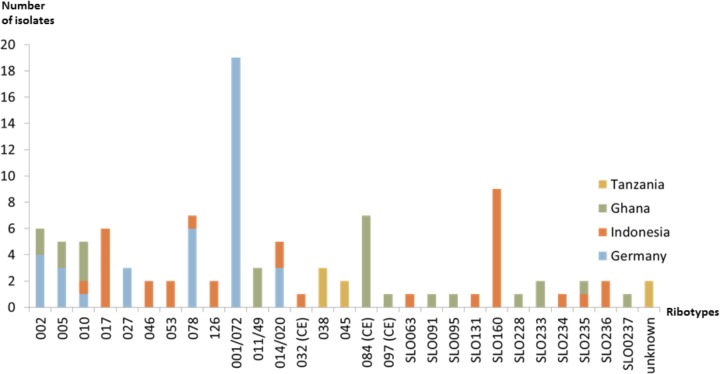
Ribotype distribution of *C. difficile* in the different study sites.

Further characterization of bacterial isolates revealed the presence of mixed infections with at least two different *C. difficile* strains only in symptomatic patients from the Indonesian study site, but not in any of the other three study sites. A total of six different ribotypes were present amongst the German *C. difficile* isolates with ribotype 001/072 dominating (62.1%, *n* = 18/29). In contrast, a broad variation of 14 different ribotypes was identified in Indonesia with SLO160 (27.3%, *n* = 9/33) and ribotype 017 (18.2%, *n* = 6/33) being most prevalent (**Figure 1**). In the Ghanaian study site, ribotype 084 was most prevalent (40.0%, *n* = 6/15) and several new ribotypes were identified amongst the total number of nine ribotypes (Janssen et al., 2016). In contrast, only three different ribotypes were present in the Tanzanian *C. difficile* isolates (Seugendo et al., 2015; **Figure 1**).

### Characterization of Toxinotypes

**FIGURE 2:**
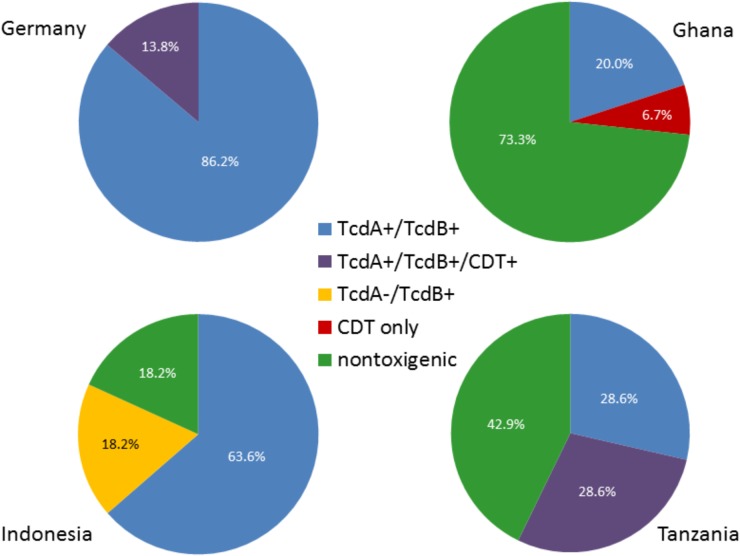
Distribution of toxinotypes in the four study sites. Percent of total numbers for the different toxinotypes in the study regions are shown. Note that rounding of percentages given for isolates from Tanzania resulted in > 100%.

As expected, all *C. difficile* isolates recovered from German patients either produced the two major toxins alone (TcdA+/TcdB+; 86.2%, *n* = 25/29) or together with the binary toxin CDT (TcdA+/TcdB+/CDT+; 13.8%, *n* = 4/29). Like in Germany, the majority of *C. difficile* from Indonesia (63.6%, *n* = 21/33) was TcdA+/TcdB+; all six ribotype 017 isolates (18.2%) were TcdA-/TcdB+, and the remaining six isolates (18.2%) were non-toxigenic (**Figure 2**). In contrast to the situation in Germany and Indonesia, a rather large ratio of African *C. difficile* isolates were non-toxigenic; 73.3% (*n* = 11/15) of the strains isolated in Ghanaian study participants and 42.9% (*n* = 3/7) of the strains recovered from Tanzanian patients (Seugendo et al., 2015; Janssen et al., 2016). In addition, one isolate from Ghana was only positive for CDT (TcdA-/TcdB-/CDT+) and two isolates from Tanzania expressed all toxins (TcdA+/TcdB+/CDT+, **Figure 2**).

### Antibiotic Susceptibility

All isolates were susceptible to metronidazole and vancomycin. The majority of 75.9% (*n* = 22/29) of *C. difficile* isolates from German patients was resistant against the macrolide erythromycin and belonged to ribotypes 001/072 (*n* = 17/18), 027 (*n* = 1/1), and 078 (*n* = 3/3). In contrast, only 15.2% (*n* = 5/33) of the isolates from Indonesia and 8/15 (53.3%) isolates from Ghana were resistant against erythromycin, including all isolates of ribotype 084 and with nearly equal distribution between symptomatic patients and asymptomatic controls (**Table 2**; Janssen et al., 2016). With 42.9% (*n* = 3/7), a similar resistance rate against the macrolide clarithromycin was found in Tanzania (Seugendo et al., 2015). With 65.5% (*n* = 19/29), an unexpectedly high percentage of the isolates from the German study site was resistant against moxifloxacin consisting of strains belonging to 001/072 (*n* = 15/18), 027 (*n* = 1/1), and 078 (*n* = 3/3). In Indonesia, only 24.2% (*n* = 8/33) of the isolates showed moxifloxacin resistance consisting mainly of ribotype 017 strains (*n* = 5/6). Moxifloxacin resistance was neither identified in Ghana nor in Tanzania.

**Table 2:** Antimicrobial resistance rates of *C. difficile* isolated in the different study sites.

Country	Metronidazole	Vancomycin	Macrolides^∗^	Moxifloxacin
Germany	0%	0%	75.9% (22/29)	65.5% (19/29)
Indonesia	0%	0%	15.2% (5/33)	24.2% (8/33)
Ghana	0%	0%	53.3% (8/15)	0%
Tanzania	0%	0%	42.9% (3/7)	0%
Total	0%	0%	45.2% (38/84)	32.1% (27/84)


*^∗^Erythromycin was tested in Germany, Indonesia, and Ghana; Clarithromycin was tested in Tanzania.*

## Discussion

Analysing a total of 1,201 inpatients with diarrhea (*n* = 608) and a control group without diarrhea (*n* = 593) in this cross-sectional multi-center study performed in a rural area of Germany and an urban area in Indonesia, and including the recently published results from a rural area of Ghana and an urban area of Tanzania (Seugendo et al., 2015; Janssen et al., 2016), we identified *C. difficile* as a likely cause of diarrhea at varying prevalence rates. Whereas *C. difficile* was present in 24 and 15% of patients suffering from diarrhea in Germany and Indonesia, respectively, this rate was significantly lower in African patients (5–6%). In addition, 1 and 5% of asymptomatic control participants from Indonesia and Ghana, respectively, were also colonized. Although age has been identified as important risk factor for colonization, *C. difficile* could neither be detected in asymptomatic control participants from Germany nor from Tanzania although most study participants from Germany were significantly older than those in Tanzania. Likewise, in contrast to the three other study sites more diarrheal patients from Germany underwent complex medical treatments and might have been prone to get hospital-acquired complications such as antibiotic-associated diarrhea caused by *C. difficile*.

Other studies revealed asymptomatic carriage rates ranging from 5–51%, which was only present in the Ghanaian, but also not in the Indonesian asymptomatic control groups (Ryan et al., 2010; Arvand et al., 2012; Alasmari et al., 2014; Galdys et al., 2014). In the Ghanaian control group, nearly all *C. difficile*-positive individuals (6/7) were colonized with non-toxigenic bacteria, which to date are considered to be non-pathogenic. On one hand, the finding that colonization was absent or a rather rare finding in our four cohorts could be attributed to the fact that our sample size was relatively small and hence the asymptomatic carriage might be underestimated. On the other hand the genesis of asymptomatic carriage remains still unclear. The different prevalence rates could also be attributed to patient-related factors, e.g., antibiotic consumption, the intestinal microbiome or nutritional habits. Recent data from Switzerland support the suggestion that asymptomatic carriage seems not to play an important role in nosocomial transmission (Pires et al., 2016).

The average age of the symptomatic *C. difficile*-positive patients varied significantly between the study sites and partly contradicted previous studies. It is widely known that advanced age and its consecutive comorbidities is one of the most important risk factors for CDI (Rupnik et al., 2009; Bauer et al., 2011; Leffler and Lamont, 2015). Indeed, in both Germany and Ghana the majority of *C. difficile*-positive patients were adults. In contrast, most patients were at child age in both Indonesia and Tanzania. A sampling bias seems to be a rather unlikely explanation for our finding because the study design, time of sampling, and microbiological methodology was identical at all study sites. Nutrition status or habits as main explanation for the age difference is unlikely, because age between *C. difficile*-positive patients differs significantly between the two African study sites. Whether *C. difficile* is affecting adults more in rural (German and Ghanaian study sites) and children more in urban environments (Indonesian and Tanzanian study sites) awaits further investigation. Data from a European surveillance study make this explanation unlikely (Bauer et al., 2011).

As it has been shown by others, the most frequently isolated ribotype in Germany was 001/072 followed by 078 (Freeman et al., 2015). In contrast, ribotype 017 was most frequently recovered from *C. difficile*-positive symptomatic patients in Indonesia. This ribotype has also been shown previously to be the most prevalent one in several Asian countries (Burke and Lamont, 2014; Knight et al., 2015). The fact that ribotype 017 *C. difficile* strains produce only TcdB has a direct impact on diagnosis of travelers or migrants from Asian countries because a false-negative status would result from toxin tests that exclusively detect TcdA only. The same is true for non-toxigenic *C. difficile* isolates; whereas 18.2% of isolates from Indonesian participants were non-toxigenic, the majority of *C. difficile* isolates from Ghanaian and Tanzanian patients and control participants were non-toxigenic. Although pathogenicity of *C. difficile* currently relies on the presence of toxins, the high prevalence of non-toxigenic isolates in Ghana, Tanzania, as well as in Indonesia awaits further investigation. Hypervirulent ribotypes 027 and 078 were only identified in a small number of patients from Germany and one patient from Indonesia, indicating that they seem to play no distinct role in our cohort of patients.

Our finding of metronidazole and vancomycin susceptibility of all *C. difficile* isolates, irrespective of origin are in line with the results of other studies, such as e.g., the European Surveillance Study (Freeman et al., 2015). In contrast to this European Surveillance Study with a moxifloxacin resistance of 48.8% for *C. difficile* strains from Germany, however, we found a high rate of moxifloxacin resistant strains (65.5%, mostly of the epidemic ribotype 001/072). This is in the range of an investigation of 34 *C. difficile* strains from Southern Germany that revealed a moxifloxacin resistance rate of 67.6% including 80% of tested ribotype 001/072 strains (Reil et al., 2012). The fact that the rate of moxifloxacin-resistant *C. difficile* isolates in Indonesia was significantly lower and resistance against this fluoroquinolone was even absent in the African study sites might mirror antibiotic consumption; whereas moxifloxacin has been introduced in Germany in 1999 and much later in Indonesia, it is still not available at the two African study sites (Brunner, 1999).

## Conclusion

This international multi-center study indicates that CDI and *C. difficile* colonization is a global health threat with geographically different prevalence rates that might reflect distinct use of antibiotics. Diagnosis should rely on glutamate-dehydrogenase testing to identify the presence of *C. difficile* and a test for determining at least TcdB production to identify toxigenic isolates.

## Author Contributions

UG had the initial idea which was developed in a project together with PC, RLK, SM, JRO, and OZ. MS, IJ, VL, IH, KG, and BO collected the samples and performed the microbiological analyses and together with OZ, WB, and UG interpreted the results. LM and MR determined ribotypes and toxinotypes. All authors wrote the manuscript and read and approved the final version.

## Conflict of Interest Statement

The authors declare that the research was conducted in the absence of any commercial or financial relationships that could be construed as a potential conflict of interest.
